# Comparative cytogenetics in three Sciaenid species (Teleostei, Perciformes): evidence of interspecific chromosomal diversification

**DOI:** 10.1186/s13039-017-0338-0

**Published:** 2017-10-23

**Authors:** Dongdong Xu, Wagner Franco Molina, Cassia Fernanda Yano, Yurong Zhang, Ezequiel Aguiar de Oliveira, Bao Lou, Marcelo de Bello Cioffi

**Affiliations:** 1 Marine Fishery Institute of Zhejiang Province, Key Lab of Mariculture and Enhancement of Zhejiang Province, 316100 Zhoushan, Zhejiang Province People’s Republic of China; 2 Departamento de Biologia Celular e Genética, Centro de Biociências, Universidade Federal do Rio Grande do Norte, Campus Universitário, Lagoa Nova, 3000, Natal, RN 59078-970 Brazil; 3 Departamento de Genética e Evolução, Universidade Federal de São Carlos, Rodovia Washington Luís, km 235, São Carlos, SP 13565-905 Brazil; 4 Secretaria de Estado de Educação de Mato Grosso – SEDUC-MT, Cuiabá, MT Brazil

**Keywords:** Sciaenidae, Chromosome evolution, Repetitive sequences, Pericentric inversions, U2 snRNA

## Abstract

**Background:**

Species belonging to the Sciaenidae family present a karyotype composed by 48 acrocentric chromosomes and are thus considered a striking example of chromosomal conservation. In this family, three species are extensively studied including *Larimichthys crocea*, *Larimichthys polyactis* and *Nibea albiflora* due to their importance in fishery and aquaculture in East Asia. Despite abundant data of population genetics available for some of them, cytogenetic information on these species is still scarce and obtained by conventional cytogenetic protocols. Therefore, a more detailed cytogenomic investigation was performed in these species to analyze their karyotype differentiation using conventional staining techniques and fluorescence in situ hybridization to map several repetitive DNAs.

**Results:**

The three species showed a slight karyotype differentiation with 4sm + 2st + 42a in *L. polyactis*, 20st + 28a in *L. crocea* and 48a in *N. albiflora*. Additionally, the mapping of repetitive sequences further revealed a number of interspecific differences among them. Particularly, 18S and 5S rDNA sites showed syntenic arrangements in *N. albiflora* and non-syntenic arrangements in both *Larimichthys* species. The microsatellites (CA)_15_ and (GA)_15_ showed conspicuous terminal clusters in some chromosomes of all species. On the other hand, (CGG)_10_ repeats, *Rex6* elements and U2 snRNA displayed a scattered distribution on the chromosomes.

**Conclusions:**

Although the three Sciaenid species examined displayed a general pattern of karyotypic conservatism, we explored chromosomal diversification among them. The diversificated karyotypic macrostructure is followed by intergeneric evolutionary diversification of the repetitive sequences. The data indicate some degree of intergeneric evolutionary diversification at chromosomal level, and suggest the evolutionary dynamics among Sciaenid species, higher than previously thought. The present cytogenetic data provide new insight into the chromosomal diversification in Sciaenidae, and contribute to inferring the chromosomal rearrangements and trends of karyotype evolution in this fish group.

## Background

**Fig. 1:**
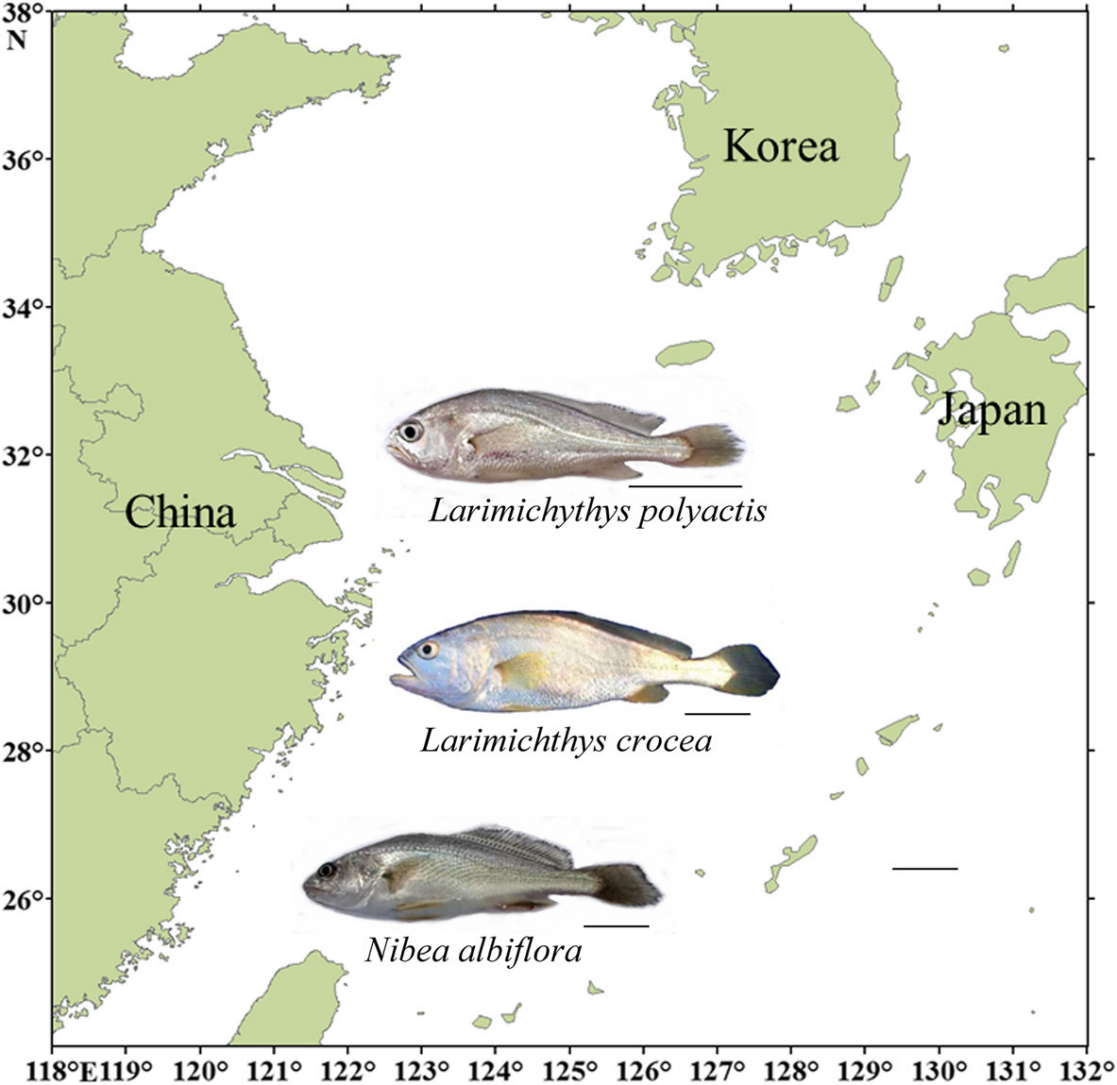
Sciaenidae species analyzed in this study and distribution regions in the China Sea. Scale bars = 5 cm

Sciaenidae form one of the largest Perciformes’ families, comprising 66 genera and approximately 291 species [1]. They are commonly called croakers or drums because of their propensity to produce sounds using sonic muscles and swim bladder. Sciaenid fishes are among the top priced seafood species in Asian cuisine due to their delicate flavor and rich nutritional value. The genera *Larimichthys* and *Nibea* include three of the most popular species in this family, which are the large yellow croaker (*Larimichthys crocea*), the small yellow croaker (*Larimichthys polyactis*) and the yellow drum (*Nibea albiflora*) [2]. They are commercially important species distributed along the coasts of East Asia (Fig. 1). In fact, *L. crocea* is one of the most highlighted aquaculture species in China, with the annual yield exceeding any other net-cage-farmed marine fish species [3], while *L. polyactis* is one of the principal marine fishery species in China and East Asian countries [4]. Phylogenetically related to *Larimichthys*, *Nibea albiflora* has great importance for commercial fisheries and is a promising candidate for aquaculture in China [5]. Together, these species have been significantly overfished, putting them at risk, therefore, over the last years initiatives have been made to preserve the wild stocks. Due to its importance, deep efforts have been applied in understanding their genetic aspects, including the analysis of population genetic structure, genetic map, and genomic prospections [3, 6–9].

In spite of the cumulative genetic information in others areas, the chromosomal data for Sciaenids are restricted to few conventional cytogenetic analyses, in which karyotypes are reported for 38 (14%) species [10]. Most of the Sciaenids examined have a diploid number (2n) of 48 chromosomes (95% of species), nearly all acrocentric (85% of species) [10, 11], revealing a remarkable example of chromosomal stability and conservation among Perciform fishes. Previous conventional cytogenetic data also showed 48 chromosomes in *L. polyactis*, *L. crocea* and *N. albiflora* [12–14]. However, the karyotype formulas of these species revealed discrepant differences, especially for *L. crocea* [14–16]. Moreover, very little is known about other important cytogenetic features, such as the incidence of chromosomal repetitive DNA elements and their evolutionary role in this fish group. Therefore, processes involved in macro-structural karyotype changes, chromosomal diversification, and genomic restructuring can be overlooked.

Molecular cytogenetics provides valuable tools and insights for comparative genomic research and has emerged as promising for understanding genome evolution and organization. In particular, the molecular organization and cytogenetic mapping of repetitive DNA elements, including satellites, multigene families and microsatellite repeats, have been analyzed in a large number of species [17]. These studies have demonstrated the enormous potential that the investigation of repetitive DNAs offers toward extending our knowledge of karyotype differentiation.

In this sense, we performed conventional (Giemsa staining and C-banding) and molecular (in situ mapping of 7 different repetitive DNAs) cytogenetic approaches in three Sciaenid species. Besides providing new cytogenetic data to this family, the present study is useful to infer the chromosomal rearrangements and trends of karyotype evolution in this fish group.

## Methods

### Materials

Fishes were sampled from the research station of Marine Fishery Institute of Zhejiang Province (Xishan Island, City of Zhoushan, China). Three species were analyzed: *Larimichthys crocea* (six males an seven females), *Larimichthys polyactis* (seven males and eight females), and *Nibea albiflora* (six males and six females).

### Mitotic chromosome preparation and chromosome banding

Mitotic chromosome preparations were obtained by the air-drying method, following Bertollo et al. (2015) [18]. The specimens were injected with 0.05% colchicine for 3 h. The kidney tissue was collected and placed in hypotonic 0.075 mol/l KCl solution for 30 min, to obtaining a cell suspension. The cells were fixed in Carnoy’s solution (methanol: acetic acid, 3: 1). Afterwards, the cells were dropped on cooled clean glass slides, air-dried and stained with 15% Giemsa solution diluted with phosphate buffer (pH 6.8). C-bands were obtained according to the method described by Sumner (1972) [19].

### Probes preparation

Two tandemly-arrayed DNA sequences isolated from the genome of an Erythrinidae fish species, *Hoplias malabaricus*, were used as probes. The first probe contained a 5S rDNA repeat copy and included 120 base pairs (bp) of the 5S rRNA transcribed gene and 200 bp of the non-transcribed spacer (NTS) sequence [20]. The second probe contained a 1400 bp segment of the 18S rRNA gene obtained via PCR from the nuclear DNA [21]. The 5S rDNA partial sequence were cloned into plasmid vectors and propagated in DH5α *E. coli* competent cells (Invitrogen, San Diego, CA, USA). The 18S and 5S rDNA probe were labeled with DIG-11-dUTP and biotin-14-dATP, respectively, by nick translation according to manufacturer’s recommendations (Invitrogen, San Diego, CA, USA).

The microsatellites d(CA)_15_, d(GA)_15_, and d(CGG)_10_ were synthesized according to Kubat et al. (2008) [22]. These sequences were directly labeled with Cy3 at 5′ terminal during synthesis by Sigma (St. Louis, MO, USA). The probes for retrotransposable element *Rex6* and the multigene family U2 snDNA were produced by PCR using primers described in Volff et al. (2001) [23] and Úbeda-Manzanaro et al. (2010) [24], respectively. Both U2 snDNA and *Rex6* probes were directly labeled with Spectrum Orange-dUTP by nick translation, according to the manufacturer’s recommendations (Roche, Mannheim, Germany).

### Fluorescence in situ hybridization and signal detection

Fluorescence in situ hybridization (FISH) experiments were performed as described in Yano et al. (2017) [25], with slight modifications. The experiment was performed under high stringency conditions on mitotic chromosome spreads. Metaphase chromosome slides were incubated with RNase (40 μg/ml) for 1.5 h at 37 °C. After denaturation of the chromosomal DNA in 70% formamide/2× SSC at 72 °C for 3 min, the hybridization mixture (2.5 ng/μl probes, 2 μg/μl salmon sperm DNA, 50% deionized formamide, 10% dextran sulphate) was dropped on the slides, and the hybridization was performed overnight at 37 °C in a moist chamber containing 2× SSC. The first post-hybridization wash was performed with 2× SSC for 5 min at 42 °C, and a final wash was performed at room temperature in 1× SSC for 5 min. The signal detection was performed using anti-digoxigenin rhodamine (Roche) for the 18S rDNA probe and with avidin-FITC (Sigma) for 5S rDNA. Subsequently, the slides were dehydrated again in an ethanol series (70%, 85% and 100%), 2 min each. Finally, the slides were counterstained with DAPI and mounted in an antifading solution (Vectashield from Vector Laboratories).

### Microscopy analyses and image processing

The chromosomes were analyzed in an epifluorescence microscope Olympus BX50 (Olympus Corporation, Ishikawa, Japan). Approximately 30 metaphase spreads were analyzed per specimen to determine the diploid chromosome number and karyotype structure. The chromosomes were classified as metacentric (m), submetacentric (sm), subtelocentric (st) or acrocentric (a) according to arm ratios (Levan et al. 1964) [26]. The count of fundamental arm number (NF) considered m, sm, and st as bi-brachial chromosomes, while terminal, as mono-brachial.

## Results

**Fig. 2:**
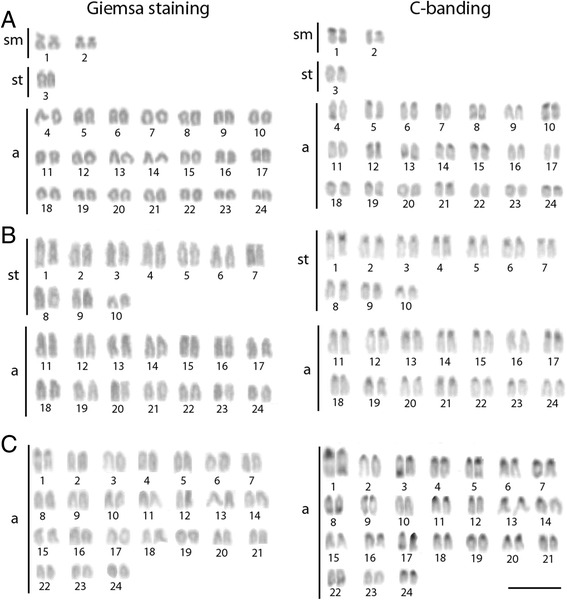
Karyotypes of *Larimichthys polyactis*, *Larimichthys crocea* and *Nibea albiflora* after Giemsa and C-banding staining. Scale bar = 5 μm

All the species presented the same diploid number (2n = 48), with karyotypes composed by 4sm + 2st +42a in *L. polyactis*, 20st + 28a in *L. crocea* and 48a in *N. albiflora* (Fig. 2). No differences between male and female karyotypes were observed in all the species. The C-positive heterochromatic blocks preferentially located in the centromeric regions, with some pairs exhibiting additional blocks in the telomeric region (Fig. 2).

**Fig. 3:**
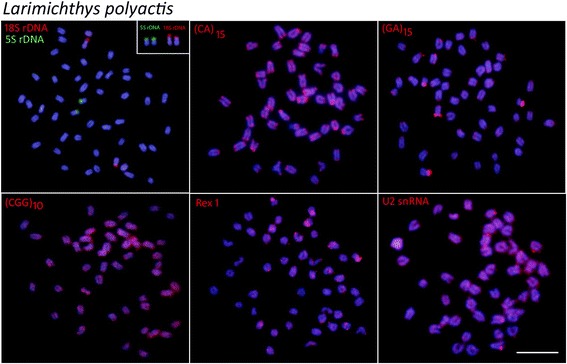
Metaphase plates of *Larimichthys polyactis* mapped with different repeated DNAs*.* 5S rDNA (green), 18S rDNA, di-and trinucleotide microsatellites, *Rex*1 and U2 snRNA (red) as probes. The chromosomes bearing 18S rDNA sites are shown in enlarged forms boxes. Scale bar = 5 μm

**Fig. 4:**
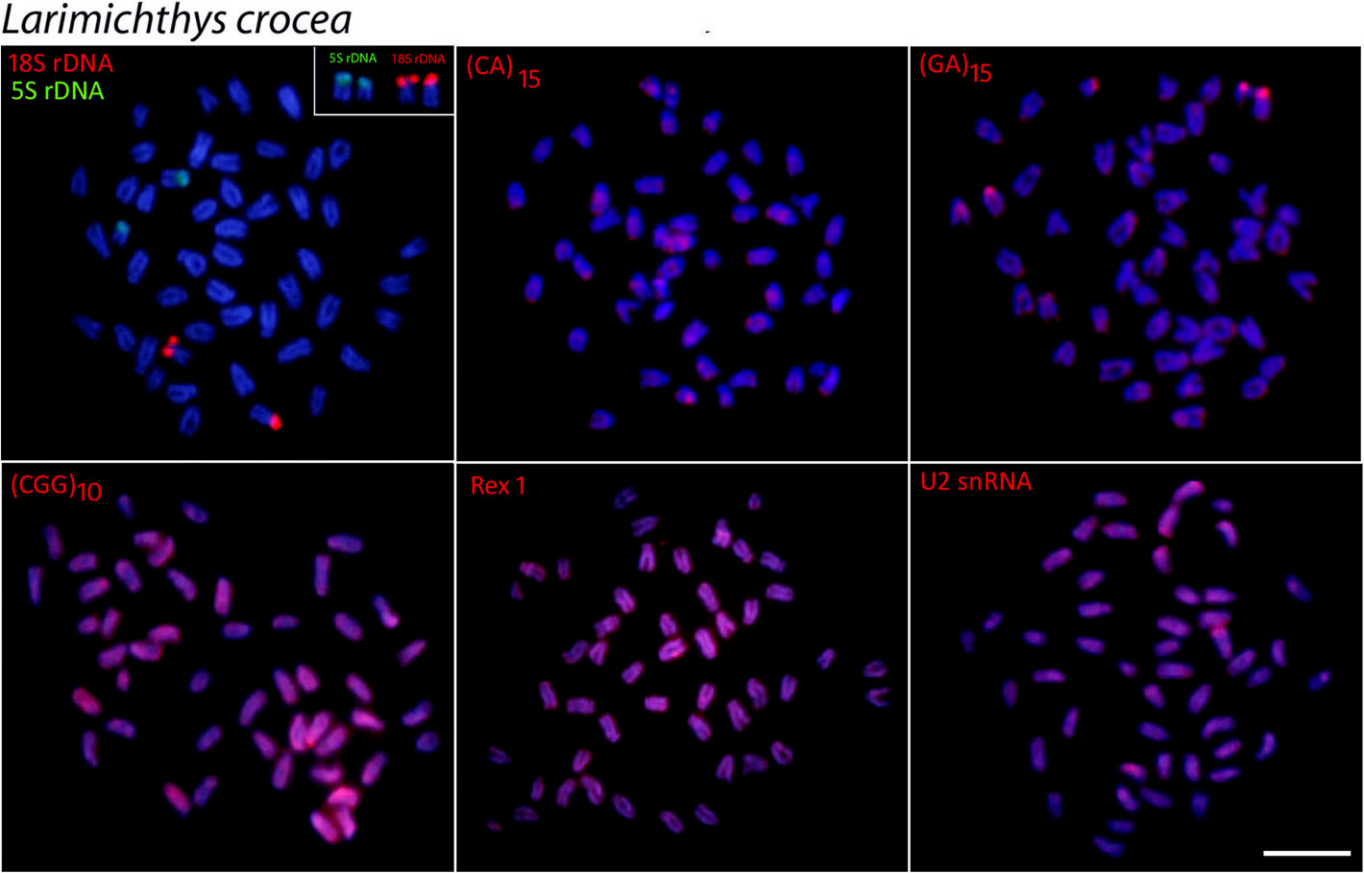
Metaphase plates of *Larimichthys crocea* mapped with different repeated DNAs*.* 5S rDNA (green), 18S rDNA, di-and trinucleotide microsatellites, *Rex*1 and U2 snRNA (red) as probes. The chromosomes bearing 18S rDNA sites are shown in enlarged forms boxes. Scale bar = 5 μm

**Fig. 5:**
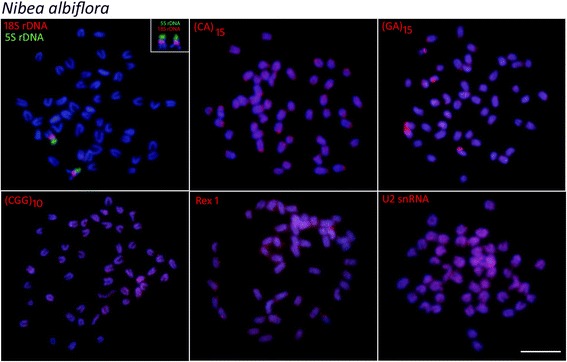
Metaphase plates of *Nibea albiflora* mapped with different repeated DNAs*.* 5S rDNA (green), 18S rDNA, di-and trinucleotide microsatellites, *Rex*1 and U2 snRNA (red) as probes. The chromosomes bearing 18S rDNA sites are shown in enlarged forms boxes. Scale bar = 5 μm

Mapping of 5S rDNA and 18 rDNA showed single sites for all the species analyzed, but exhibited different distribution patterns. In *L. polyactis* and *L. crocea,* both genes were located in the terminal position of short arms of two distinct sm chromosomal pairs (Figs. 3 and 4). On the other hand, in *N. albiflora* the 18S rDNA and 5S rDNA sites have a syntenic organization, with the 18S rDNA sequences located in the terminal region of the short arms and the 5S rDNA cluster in the proximal region of the long arms of an acrocentric chromosome pair (Fig. 5).

The chromosomal mapping of microsatellites (CA)_15_ and (GA)_15_ showed similar patterns for all species, with a remarkable accumulation in the subtelomeric regions of some chromosomes. However, different patterns were observed among species with (CGG)_10_, since, in addition to the presence of scattered signals, a considerable accumulation of this sequence was detected in both *Larimichthys* species (Figs. 3 and 4); while very weak and disperse signals were observed in the chromosomes of *N. albiflora* (Fig. 5). FISH using the PCR fragments of *Rex6* and U2 snDNA, presented a similar dispersion pattern in which the signals were abundantly distributed in the chromosomes of all the species (Figs. 3, 4, 5).

## Discussion

### Karyotype differentiation

In the present study, we characterized the karyotypes and chromosomal features of three croaker fishes using conventional and molecular cytogenetic approaches. To our knowledge, these are the first molecular cytogenetic data for any Sciaenid species.

The karyotypes for the three fish species exhibited basal chromosome number (2n = 48) and predominance of acrocentric chromosomes. The results are basically consistent with that reported in previous Giemsa staining analyses. However, different chromosomal formulas were reported for *L. crocea* and *L. polyactis* in previous studies. In *L. crocea*, the cryptic classification among the subtelocentric and acrocentric chromosomes has caused divergences in the chromosomal formulas reported by different authors [15, 16]. However, the cytogenetic analysis in *L. polyactis* reveals a yelling karyotype discrepancy with previous studies in this species*.* In fact, our study showed the presence of three bi-brachial chromosome pairs not identified in previous reports [12]. The detection of discrete chromosome differences in *L. crocea* and more conspicuous in *L. polyactis* may show evidence of polymorphism in these species. Nevertheless, the small chromosomal size and the lack of notable technical improvement in fish chromosome obtainment and analysis not rule out the possibility of chromosomal artifacts. Our study is a step forward to clarifying these issues and understanding the biodiversity of marine Sciaenidae.

Sciaenidae is characterized by a symmetrical and conservative karyotypic pattern, with most of them displaying 2n = 48 and karyotypes with a high number of acrocentric chromosomes [10]. This conservative pattern is recurrent in Atlantic species analyzed [11]. Similarly, the three species analyzed also displayed the conserved karyotype observed for most marine fishes, especially in *N. albiflora* which is a remarkable example with a chromosome set made up of 48 acrocentric chromosomes. Nevertheless, significant karyotypic differentiations were observed in genus *Larimichthys*. Indeed, *L. polyactis* shows two sm chromosomes pairs and one pair of st chromosomes (NF = 54), while *L. crocea* possesses 10 pairs of st (NF = 68). Such variations in chromosomal morphology indicate that pericentric inversions, like in other Perciformes [27], are the primary mechanisms for karyotype diversification among them.

### Chromosomal distribution of repetitive sequences

The in situ localization and chromosome mapping of seven classes of repetitive DNA sequences resulted in useful characteristics for comparative genomics at chromosomal level. Among these DNA classes, the ribosomal RNA genes are the most mapped sequences for purposes of chromosomal evolution analysis in fishes. Accordingly, chromosomal distribution of rDNA clusters can be informative for comparative analysis of closely related species or even to the characterization of population variations.

The 18S rDNA sites were located on the terminal position in a sm chromosome pair of *L. polyactis* and in a st chromosome pair of *L. crocea*, suggesting that rearrangements involving 18S rDNA loci in the homologous chromosome occurred during the divergence of these congeneric species of *Larimichthys*. The 5S and 18S rDNA sites display non-syntenic arrangements in the genus *Larimichthys*, while notably, these genes occur in a syntenic array in *N. albiflora*. The non-syntenic arrangements of 5S and 18S rDNA loci are the most common features in chromosomes of fishes, indicating independent evolution of these loci [28, 29]. On the contrary, the syntenic association of 5S and 18S rDNA sites evidenced in *N. albiflora* is infrequent in fishes, despite these gene arrays have been reported to several species among them [30, 31]. Taken together, the different syntenic patterns of 5S and 18S rDNA sites found here suggest that these regions may represent an important feature for karyotype diversification in the three Sciaenides.

Some repetitive sequences, especially the microsatellites, are particularly variable in fish chromosomes and linked to karyotype diversification. In the analyzed species, microsatellites (CGG)_10_ were strongly distributed in the chromosomes of *Larimichthys*, but with a distinct and more discrete distribution pattern in *N. albiflora*. The organization of (CGG)_10_ in fish chromosomes have demonstrated that these repeat motifs can show particular arrangements among different species [17]. Since these sequences are subjected to high rates of changes, their distribution indicate distinct evolutionary pathways concerning the genome organization among genus *Larimichthys* and *Nibea*.

*Rex* is comprised of various families of transposable elements that are abundant in teleosts, and their distribution varies from a scattered pattern to preferential accumulation in certain regions of the chromosomes [32]. Our data show that *Rex*1 elements display a diffuse and sparse distribution, as similarly described for some fishes, as in naked catfish *Mystus bocourti* [33], cichlids [34] and several Atlantic snappers [35]. Generally this distribution pattern is nonrandom and seems to have some relation to specific characteristics of sub-regions of the host genomes, indicating that TEs are important structural components of the heterochromatic regions and have played an important role in dynamic processes of fish karyotype evolution.

The multi-gene family U2 snRNA, which is one of the components of the small nuclear ribonucleoprotein particles (snDNA) and responsible for mRNA splicing, exhibited a very widely scattered pattern in the three analyzed species, with intense hybridization signals in some specific regions. This genomic organization of the three species is quite similar to the one found for the fishes *Amphichthys cryptocentrus*, *Porichthys plectrodon* and *Abudefduf* species [24, 36]. Contrasted with the aforementioned results, most of the data available for different groups showed that these conserved genes are organized in a single or a small number of chromosomal clusters [33]. It has been proposed a trend for the U2 snRNA genes to accumulate in a specific chromosome pair over the course of the evolutionary history in the Batrachoididae family [24]. It would be interesting to study this gene in more members of Sciaenid family to deduce its distribution trend in the chromosomes.

### Chromosomal differentiation and speciation

Sciaenid fishes displayed extensive phenotypic diversity, especially for the species distributed in Eastern Pacific. However, the speciation process is not considered to be followed by significant karyotype differentiation [11, 15]. In the present study, significant karyotype and macro-karyotype differentiation were explored among the three Schianid species, allowing a connection between the chromosomal data and the biogeographic events related to this group of fish. It is hypothesized that historical environmental variations caused by glacial events gave rise to the initial diversification in this family [37], leading to population restrictions and the fixation of genetic divergences, such as the chromosomal arrangements. The role of glacial events in fractioning and restricting population is considered relevant for the chromosomal diversification and transitory polymorphisms present in marine species of the genus *Chromis* [38].

It is noteworthy that *L. polyactis* and *L. crocea* share morphological characteristics [2] and have similar mtDNA barcodes [4]. Molecular analyses show that these two congeneric species are closely related, with divergence estimated at around 4.62 Mya [37], suggesting a recent sympatric speciation. The cytogenetic divergences promoted by pericentric inversions could lead to the effective postzygotic reproductive isolation between both species during that time. However, wild stocks of genus *Larimichthys* were severely depleted, since they have been extensively explored in the past decades, especially *L. crocea* [16]. These anthropogenic factors lead to the changes in life history and expansion of the distribution area, and thereby promoting hybridization events as demonstrated in various marine species [39]. These conditions are particularly worrisome in *Larimichthys* because interspecific breeding between *L. polyactis* and *L. crocea* were found to produce viable hybrid offspring (data unpublished). The potential fragility of the reproductive isolation mechanisms among *Larimichthys* species and their considerable degree of chromosomal similarity promote low protection to ensure the preservation of the genetic cohesion of the species. Ineffective reproductive blocking may cause reverse speciation, a condition that enables the mixture or the genetic submersion of the gene pool of a species into another [40]. In this sense, extending analyses are required to clarify the hybrid events between *L. polyactis* and *L. crocea*.

## Conclusions

Sciaenid fishes are valuable in Asian fishery and aquaculture, especially the species analyzed in the present study. Our results showed significant interspecific chromosomal diversification among the three Sciaenid fishes examined, incorporated a general pattern of karyotypic conservatism. *N. albiflora* is characterized by a basal karyotype, while *Larimichthys* shows karyotype diversifications promoted by pericentric inversions. Moreover, such variability is also reinforced by the dynamism of repetitive elements in the genome, especially by the differential distribution and accumulation of rDNA sequences among chromosomes.

The karyotype divergences between *L. polyactis* and *L. crocea* could act as post-zygotic chromosomal barriers. On the other hand, these species showed a remarkable similarity of chromosomal organization of repetitive sequences and functional genes. Thus, the anthropological activities such as overfishing, stock enhancement, and changes in marine environment could breakdown the “calm sea of speciation”. In this aspect, extending cytogenetic and molecular studies are relevant to provide informative data for diagnosing possible interspecific hybridization and introgression between them.
